# Descriptions of new species of the genera *Sarima* Melichar and *Sarimodes* Matsumura from southern China (Hemiptera, Fulgoromorpha, Issidae)

**DOI:** 10.3897/zookeys.557.6166

**Published:** 2016-01-28

**Authors:** Rui Meng, Yinglun Wang

**Affiliations:** 1 Key Laboratory of Plant Protection Resources and Pest Management of the Ministry of Education; Entomological Museum, Northwest A&F University, Yangling, Shaanxi 712100, China

**Keywords:** Fulgoroidea, morphology, taxonomy, checklist, Hainan Island, Yunnan

## Abstract

Two Issini genera, *Sarima* Melichar, 1903 and *Sarimodes* Matsumura, 1916, are examined. One new *Sarima* species: *Sarima bifurcus* **sp. n.** and two new *Sarimodes* species *Sarimodes clavatus* **sp. n.** and *Sarimodes parallelus* **sp. n.** are added from South China. A checklist of species in the genus *Sarima* with data on distribution is provided. The distribution and morphological peculiarities of the genera *Sarima* and *Sarimodes* are briefly discussed.

## Introduction

The genus *Sarima* belongs to the Issini Spinola, 1839 and was erected by Melichar (1903) for two species from Sri Lanka: *Sarima illibata* Melichar, 1903 (type species) and *Sarima elongata* Melichar, 1903. Subsequently, Distant (1906) recognized the genus and described one species *Sarima cretata* from Sri Lanka. Meanwhile, Melichar (1906) described eight species (*Sarima castanea*, *Sarima nigroclypeata*, *Sarima separata*, *Sarima solia*, *Sarima amagisana*, *Sarima notata*, *Sarima bimaculata* and *Sarima clathrata*), and two other species: *Hysteropterum subsfasciata* Melichar, 1903 and *Hysteropterum fuscula* Melichar, 1903 were transferred to this genus. Distant (1909), Schmidt (1910, 1928), Schumacher (1915), Jacobi (1928, 1944), Esaki (1931), Kato (1933), Matsumura (1916, 1936), and Fennah (1950) subsequently added 16 species to the genus (Metcalf 1958). Three more species were described by Hori (1970, 1971) respectively from Japan and the Philippines. *Sarima yohenai* Matsumura 1936 was regarded as a junior synonym of *Sarima satsumana* Matsumura 1916 by Hori (1970). Chan and Yang (1994) transferred *Sarima matsumurai* Esaki, 1931 and *Sarima rubricans* Matsumura, 1916 to *Eusarima* Yang, 1994. Recently, Gnezdilov (2013a) erected a genus *Pavelauterum* for the species *Sarima fuscula* (Melichar, 1903). Gnezdilov (2013b) transferred six species to the genus *Eusarima* Yang: *Sarima formosana* Schumacher, 1915, *Sarima koshunensis* Matsumura, 1916, *Sarima kuyaniana* Matsumura, 1916, *Sarima rinkihonis* Matsumura, 1916, *Sarima satsumana* Matsumura, 1916, and *Sarima versicolor* Kato, 1933. Currently, 22 species are included in the genus *Sarima* from the Eastern Palaearctic, Oriental, and Australian Regions (Metcalf 1958, Hori 1970, 1971, Gnezdilov 2013a, 2013b, Bourgoin 2015).

The Issini genus *Sarimodes* was erected by Mastumura (1916) for the single species *Sarimodes taimokko* from Taiwan. Recently, Gnezdilov and Hayashi (2013) suggested *Paravindilis* Yang, 1994 as a junior synonym of *Sarimodes* Matsumura based on photos of the holotype female of *Sarimodes taimokko* (Gnezdilov and Hayashi 2013) available on the web-site of the Hokkaido University as well as the illustration of Chan and Yang (1994). Meanwhile, *Pterilia formosana* and *Paravindilis taiwana* Yang, 1994 were both designated as junior synonyms of *Sarimodes taimokko*, and *Paravindilis taiwanensis* was proven to be an invalid name (Gnezdilov and Hayashi 2013). So far, the genus *Sarimodes* has only one known species distributed in Taiwan.

In this paper, one new species of *Sarima* and two new species of *Sarimodes* are described. A checklist of *Sarima* species with data on their distribution is provided below.

## Materials and methods

External morphology was observed under a Leica MZ 125 microscope. All measurements are given in millimeters (mm). Terminology used for the external morphology and the male genitalia mainly follows Chan and Yang (1994). The description of the female genitalia follows Bourgoin (1993) and Gnezdilov et al. (2014), and forewing venation pattern follows Bourgoin et al. (2015). The genital segments of the examined specimens were dissected and macerated in hot 10% NaOH solution for approximately 2–3 minutes, and subsequently transferred into glycerin. Photographs of the specimens were made using a Leica M205A microscope with a Leica DFC Camera. Images were produced using the software version LAS (Leica Application Suite) V3.7. All specimens studied are deposited in the Entomological Museum of Northwest Agriculture and Forestry University (NWAFU), Yangling, China.

## Taxonomy

### Family Issidae Spinola, 1839 Subfamily Issinae Spinola, 1839 Tribe Issini Spinola, 1839

#### Sarima

Taxon classificationAnimaliaHemipteraIssidae

Melichar, 1903

Sarima Melichar, 1903: 78. Type species: *Sarima illibata* Melichar, 1903, by original designation.

##### Diagnostic characters.

The genus *Sarima* was originally described by Melichar (1903), subsequently designated by Distant (1906) and recently redescribed by Gnezdilov (2013a). It can be distinguished from other genera in the tribe Issini by frons enlarged above clypeus, sublateral carinae distinct only in upper half of frons; ocelli present; tegmen elongate, with hypocostal plate, veins ScP + R dividing near to the basal cell, ScP short and fusing with R and forming a loop (Fig. 3, see the arrow), MP with three branches (MP dividing beyond middle of tegmen, and MP1 dividing in distal half of wing), CuA bifurcate (dividing near wing mid-point); clavus as long as nearly 4/5 of wing length; Pcu and A1 joint at mid-point of clavus. Hind wing three-lobed. Hind tibia with two lateral spines in its distal half and with 6–7 apical spines.

**Figures 1–3.**
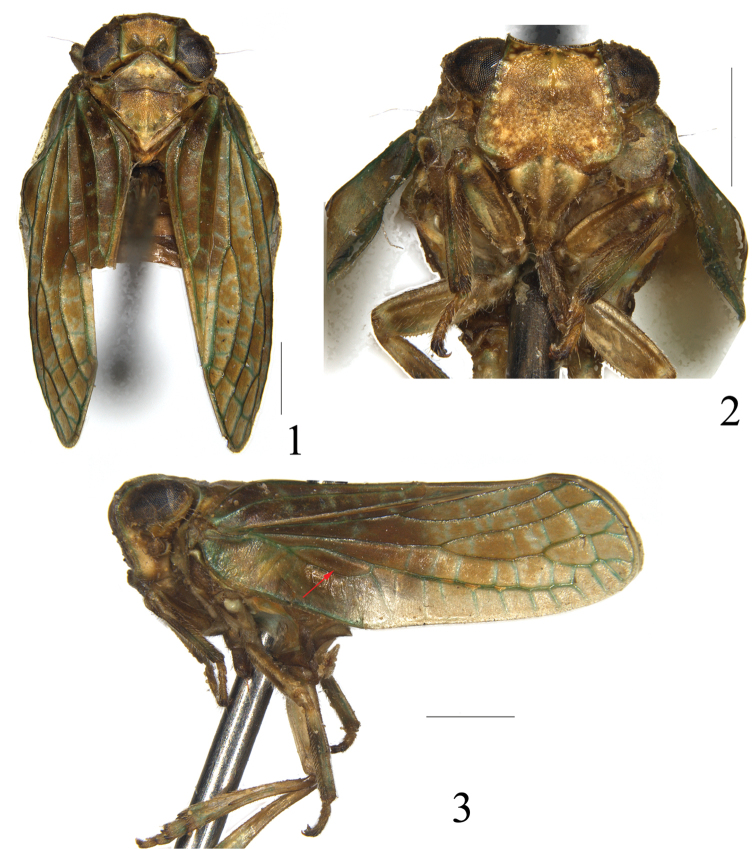
*Sarima bifurcus* sp. n. **1** adult, dorsal view **2** frons and clypeus **3** adult, lateral view. Scale bars: 1 mm.

##### Checklist of *Sarima* species

*Sarima amagisana* Melichar, 1906 – Indonesia (Sumatra, Java), Japan

*Sarima bifurcus* sp. n. – China (Yunnan)

*Sarima bimaculata* Melichar, 1906 – New Guinea

*Sarima carinata* Schmidt, 1910 – Indonesia (Sumatra)

*Sarima castanea* Melichar, 1906 – Philippines (Luzon)

*Sarima clathrata* Melichar, 1906 – Malaysia

*Sarima cretata* Distant, 1906 – Sri Lanka

*Sarima elongata* Melichar, 1903 – Sri Lanka (Gnezdilov 2013a: figs 6, 10, 11)

*Sarima erythrocyclos* Fennah, 1950 – Fiji

*Sarima illibata* Melichar, 1903 (type species) – Sri Lanka (Melichar 1906: fig. 73, Distant 1906: fig. 174, Gnezdilov 2013a: figs 1, 3, 5, 8, 9)

*Sarima miyatakei* Hori, 1971 – Philippines (Hori 1971: figs 10–15)

*Sarima nigrifacies* Jacobi, 1944 – China (Fujian)

*Sarima nigriventris* Schmidt, 1928 – Indonesia (Java)

*Sarima nigroclypeata* Melichar, 1906 – India

*Sarima notata* Melichar, 1906 – New Guinea

*Sarima novaehollandiae* Jacobi, 1928 – Australia (Queensland) (Gnezdilov and Fletcher 2010: fig. 13)

*Sarima palawana* Hori, 1971 – Philippines (Hori 1971: figs 1–9)

*Sarima ryukyuana* Hori, 1970 – Japan (Ryukyus) (Hori 1970: figs 11–17)

*Sarima separata* Melichar, 1906 – Indonesia (Mentawai, Sipora)

*Sarima sinensis* (Walker, 1851) – China (Hong Kong)

*Sarima solita* Melichar, 1906 – Malaysia

*Sarima subfasciata* (Melichar, 1903) – Sri Lanka

*Sarima tappana* Matsumura, 1916 – China (Taiwan), Japan

#### Sarima bifurcus sp. n.

Taxon classificationAnimaliaHemipteraIssidae

http://zoobank.org/DFDB3CAE-384E-4514-9102-BEE03F333763

Figs 1–3 , 4–9 , 10–16

##### Type material.

Holotype: male, China: Yunnan, Mengla County, Yaoqu Town, 6 May 1991, coll. Yinglun Wang, Wanzhi Cai; Paratypes: 1 female, same data as holotype; 1 female, China: Yunnan, Menghai County, 25 October 1987, coll. Jinian Feng, Yonghui Cai.

##### Diagnosis.

This species is similar to *Sarima ryukyuana* (Hori 1970: Figs 11–17) but differs from the latter by: 1) generally dark brown alternated with green, in *Sarima ryukyuana*, general coloration brown with dark patches; 2) pygofer with hind margin strongly convex, in *Sarima ryukyuana*, pygofer with hind margin faintly rounded; 3) aedeagus with long process reaching to basal 1/3, the process bifurcated apically in ventral view, in *Sarima ryukyuana*, aedeagus with long process reaching to base, the process bifurcated basally in lateral view.

##### Description.

Male length (n = 1) (including tegmen): 6.2 mm, length of tegmen: 4.8–4.9 mm; female length (n = 2) (including tegmen): 6.3–6.5 mm, length of tegmen: 5.5–4.6 mm.

***Coloration.*** Generally dark brown alternated with green. Eyes dark brown. Frons pale brown with yellow brown tubercles, and green near lateral margins. Clypeus brown with median carina and lateral sides yellowish brown. Ocelli brown. Gena yellow with inconspicuous dark speckles. Tegmen dark brown, longitudinal and transverse veins green. Hind wing pale brown with brown to black veins. Leg brown, apex of fore femora and base of fore tibia with dark brown. Abdomen ventrally pale yellowish green and dorsally dark brown, apex of each segment slightly pale yellowish green (Figs 1–3).

**Figures 4–9.**
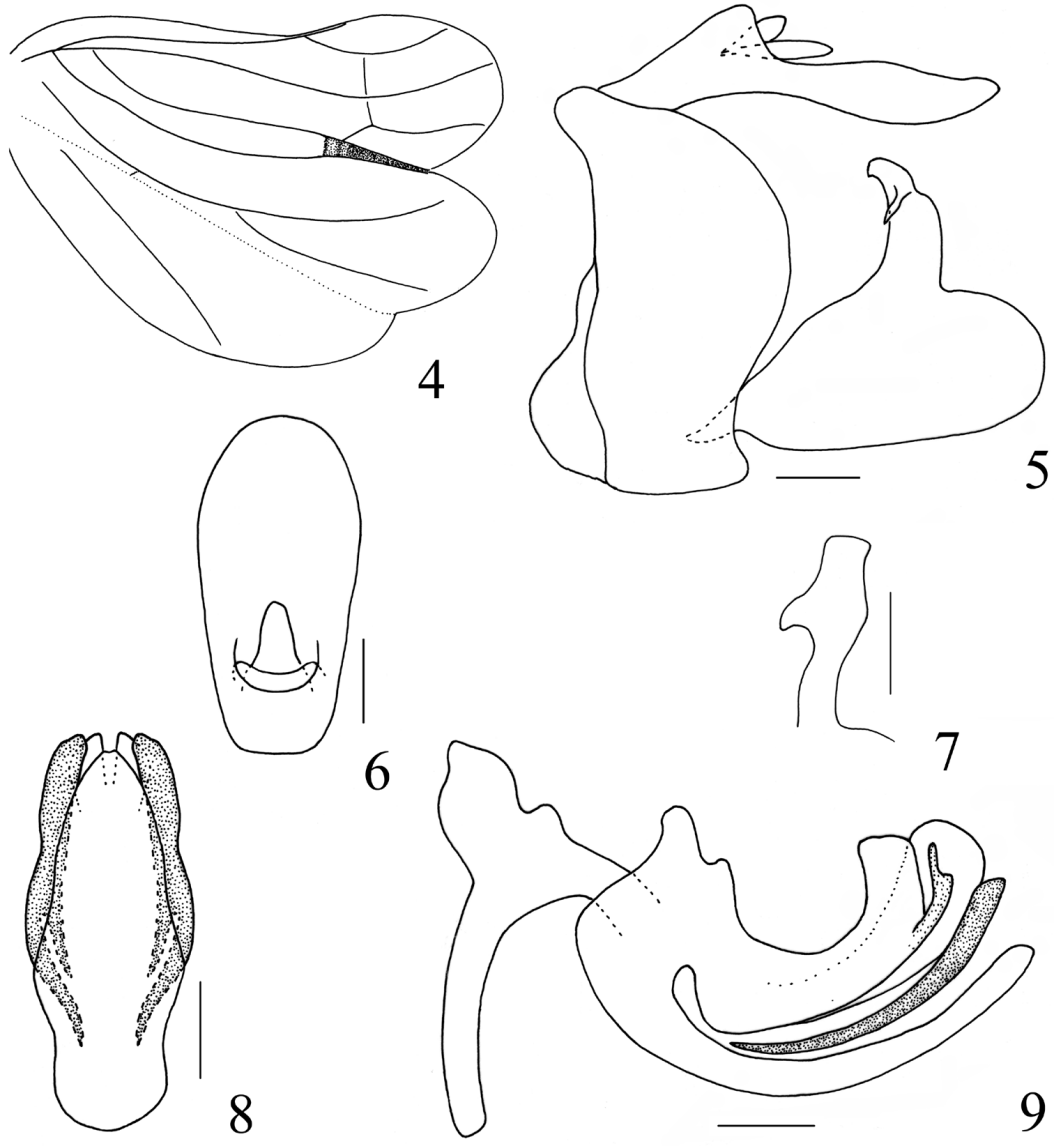
*Sarima bifurcus* sp. n. **4** hind wing **5** male genitalia, lateral view **6** male anal segment, dorsal view **7** capitulum, dorsal view **8** phallus, ventral view **9** phallus, left view. Scale bars: 0.2 mm.

***Head and thorax.*** Vertex nearly hexagonal, disc distinctly depressed, with median carina and two round depressions at disc, anterior margin angularly convex and hind margin concave, margins carinated, 1.8 times wider at apex than length in midline (Fig. 1). Frons coarse with small punctures, disc slightly elevated and distinctly expanding below antennae, with median carina and lateral carinae only distinct at upper half of frons; frons with tubercules along lateral margins and upper margin, 0.8 times longer than widest part, 1.8 times wider at widest part than at base (Fig. 2). Frontoclypeal suture distinctly curved. Clypeus smooth with median carina (Fig. 2). Pronotum with anterior margin strongly acutely convex, hind margin nearly straight, disc with median carina and two small pits (Fig. 1); paranotal lobe relatively small, lateroventral angle rounded (Fig. 2). Mesonotum subtriangular with median carina, two small depressions along lateral margin, 2.3 times wider at widest part than long in midline (Fig. 1). Tegmen subquadrate, anterior margin nearly parallel to sutural margin, longer than wide, 2.4 times longer than widest part (Fig. 3). Hind wing with R bifurcate, M, CuP, Pcu, A1 and A2 simple, CuA trifurcate; R2 and M and between M and CuA1 both with single transverse vein almost in a straight line, CuA3 and CuP fused and thickened (Fig. 4). Metatibiotarsal formula 2+7/9/2.

**Figures 10–16.**
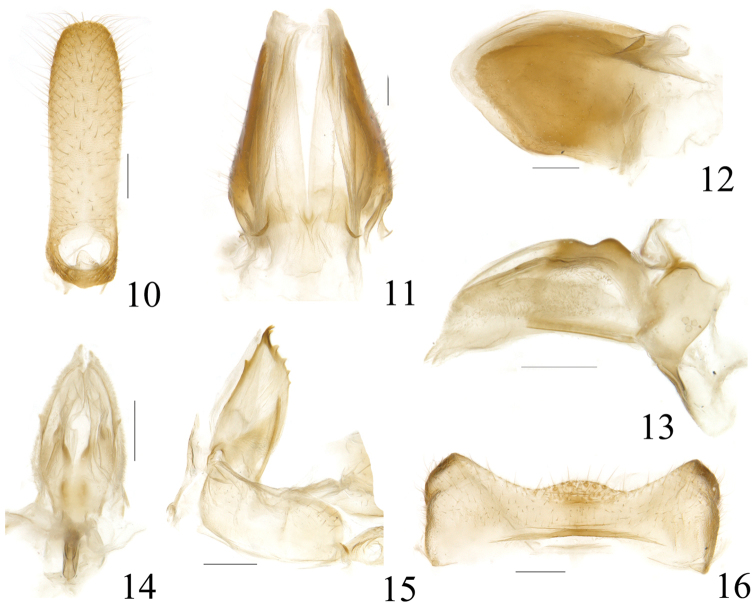
*Sarima bifurcus* sp. n. **10** female anal segment, dorsal view **11** gonoplac, dorsal view **12** gonoplac, right view **13** gonapophyses IX and gonaspiculum bridge, right view **14** gonapophyses IX and gonaspiculum bridge, dorsal view **15** gonocoxa VIII and gonapophysis VIII, right view **16** sternum VII, ventral view. Scale bars: 0.2 mm.

**Figures 17–19.**
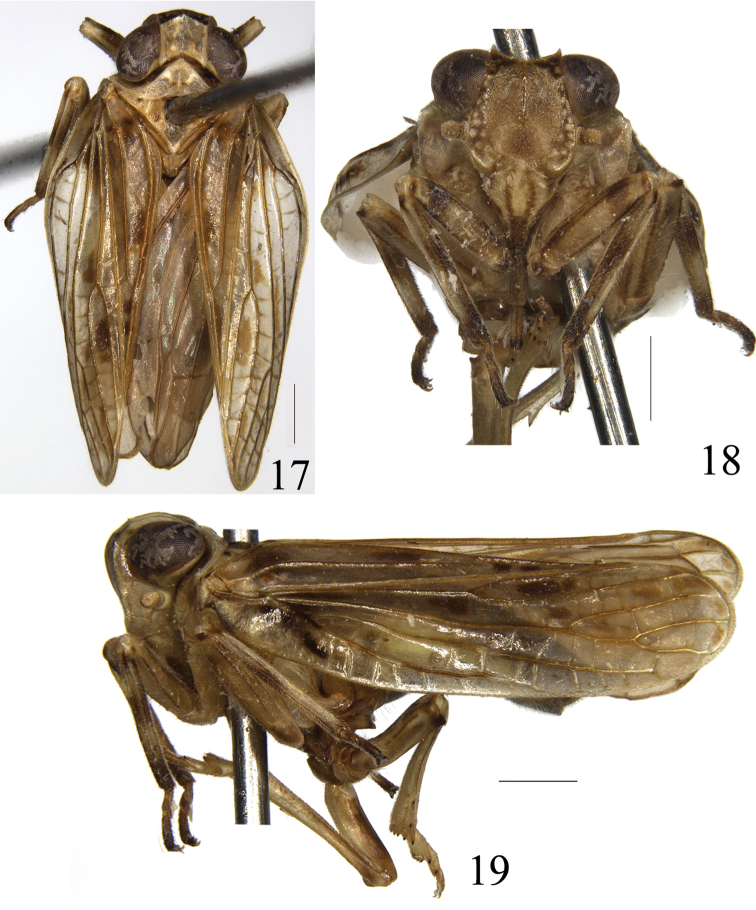
*Sarimodes clavatus* sp. n. **17** adult, dorsal view **18** frons and clypeus **19** adult, lateral view. Scale bars: 1 mm.

***Male terminalia.*** Anal segment in dorsal view nearly oval, widest near apex, apical margin obtusely convex; anus situated at basal part (Fig. 6). Pygofer with hind margin obtusely produced at dorsal half, and slightly concave near ventral margin (Fig. 5). Phallobase with dorsolateral lobe split near apex, lateral lobe forming a small short process near apex, abruptly tapered apically; ventral lobe split from dorsolateral lobe at base, gradually narrowing to apex, apical margin weakly concave at middle in ventral view; aedeagus with long process arising from apex to basal 1/3, the process bifurcated near its apex in ventral view, the inside branch slightly shorter than half length of the outside one (Figs 8, 9). Genital style in lateral view subtrianglar, with hind margin strongly concave, caudoventral angle roundly convex (Fig. 5). Capitulum elongate, basal half thin and widened at middle, with a small lateral tooth (Fig. 7).

***Female terminalia.*** Anal segment in dorsal view suboblong, elongate, lateral margins nearly parallel, weakly widened at subapex, apical margin slightly convex; anus short, situated at base (Fig. 10). Gonoplac elongate, with wide membranes near apex, apical margin strongly convex at dorsal half, disc elevated near base in dorsal view, in dorsal view fork faintly pigmented (Figs 11, 12). Proximal part of posterior connective lamina of gonapophyses IX strongly convex in lateral view, median field bifurcate at apex in dorsal view, lateral fields with a pair of short teeth near middle, with the surface bearing numerous microvilli (Figs 13, 14). Anterior connective laminae of gonapophysis VIII broad, ventral margin straight, bearing two small teeth near apex, apical group with three small similar-sized of teeth, with four teeth in lateral group (Fig. 15). Sternum VII with apical margin distinctly arcuately convex at middle (Fig. 16).

##### Etymology.

The specific epithet is derived from the Latin word “*bifurcus*”, referring to the bifurcated process of the aedeagus in ventral view.

#### Sarimodes

Taxon classificationAnimaliaHemipteraIssidae

Matsumura, 1916

Sarimodes Matsumura, 1916: 115. Type species: *Sarimodes taimokko* Matsumura, 1916.
Paravindilis Yang, 1994: 94 (in Chan and Yang 1994). Type species: *Paravindilis taiwana* Yang, 1994. Synonymised by Gnezdilov and Hayashi 2013.

##### Diagnostic characters.

The distinctive characters used by Matsumura (1916) are modified as follows.

**Figures 20–25.**
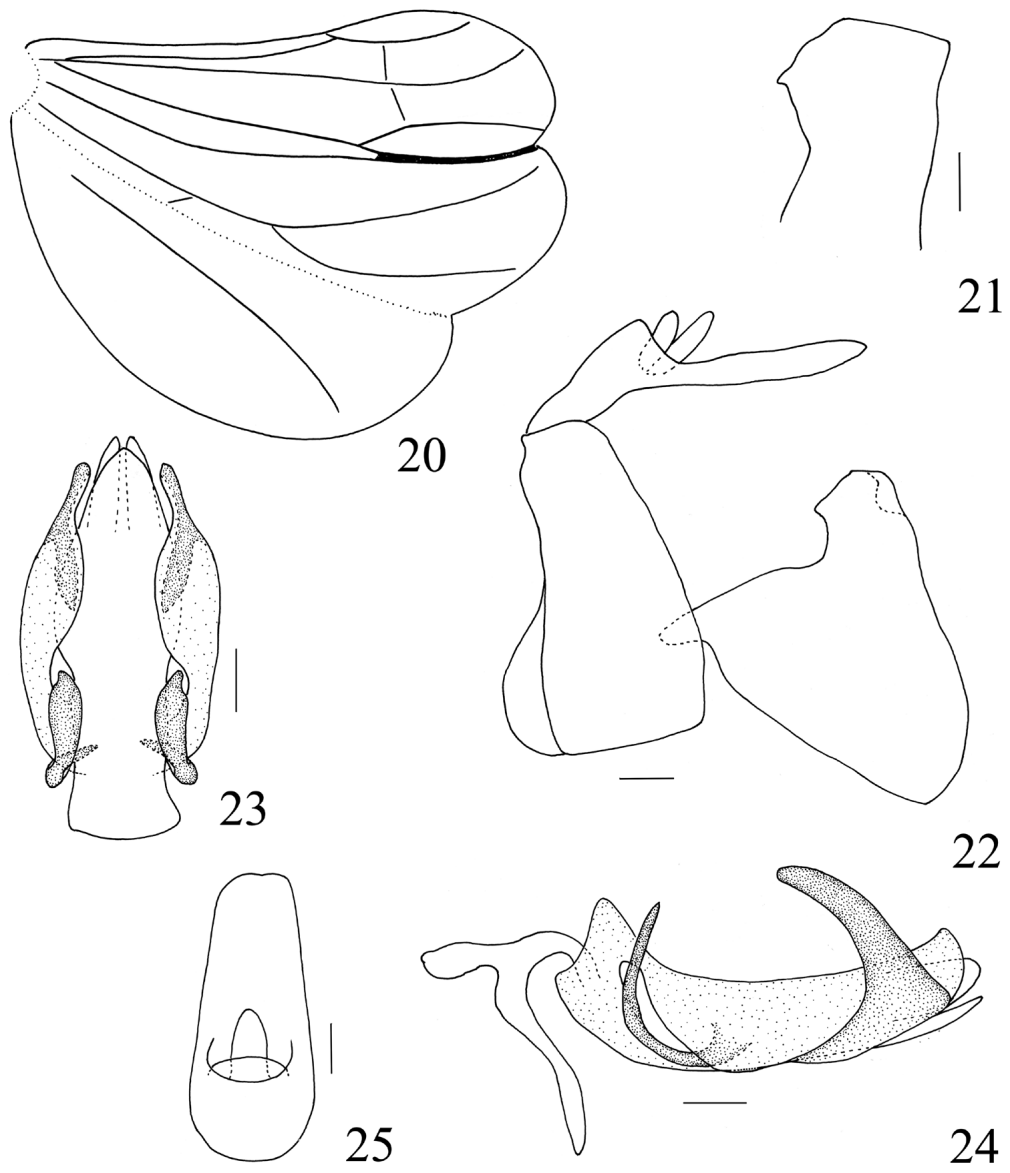
*Sarimodes clavatus* sp. n. **20** hind wing **21** capitulum, dorsal view **22** male genitalia, lateral view **23** phallus, ventral view **24** phallus, left view **25** male anal segment, dorsal view. Scale bars: 0.2 mm.

Head with eyes slightly narrower than pronotum. Vertex hexagonal, all margins ridged, with weak median carina, disc moderately depressed (Figs 17, 33). Frons slightly longer than wide, upper margin distinctly concave, lateral margins ridged and diverging to below level of antennae thence incurved to frontoclypeal suture, disc convex in upper half, with a row of submarginal tubercules laterally, with short median carina, sublateral carina indistinct (Figs 18, 34). Ocelli present. Frontoclypeal suture arcuately curved upward (Figs 18, 34). Clypeus with disc slightly convex. Rostrum reaching post-trochanter. Pronotum almost as long as vertex, with anterior margin acutely convex, posterior margin nearly straight, median carina present, with two central pits (Figs 17, 33). Mesonotum moderately shorter than pronotum and vertex combined in middle line, with three carinae (Figs 17, 33). Tegmen without hypocostal plate, costal margin convex near basal one-fourth of tegmen, narrowing to obtuse apical margin, longitudinal veins distinctly prominent and transverse veins relatively weak, ScP+R forking near basal cell, ScP just reaching or a little beyond midlength of tegmen, MP forked near distal one-third of tegmen, MP1 bifurcate near apex, CuA forked near middle, almost at the same point as the union of claval veins; clavus almost extended to apical margin (Figs 19, 35). Hind wing well developed, trilobed, veins R, M, CuP, Pcu, A1 and A2 simple, CuA bifurcate, CuA2 and CuP fused and thickened (Figs 20, 36). Hind tibia with two lateral teeth and seven spines apically.

**Figures 26–32.**
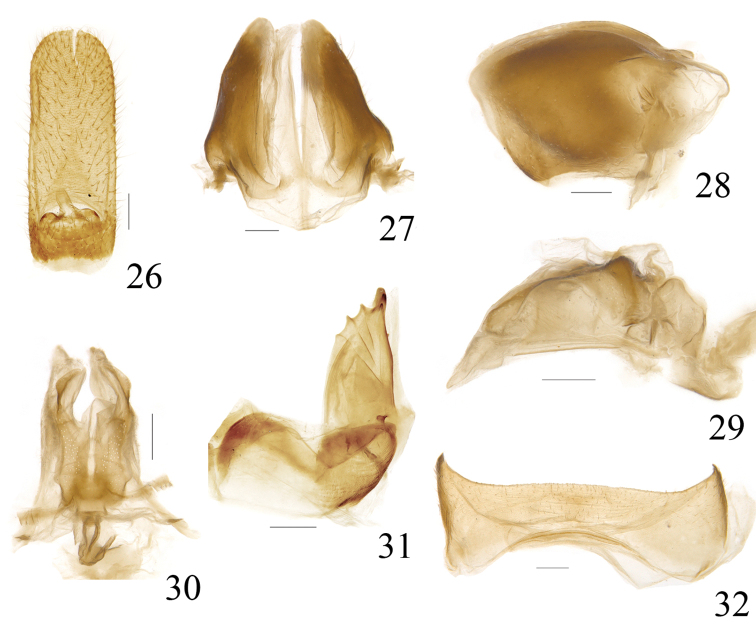
*Sarimodes clavatus* sp. n. **26** female anal segment, dorsal view; **27** gonoplac, dorsal view **28** gonoplac, right view **29** gonapophyses IX and gonaspiculum bridge, right view **30** gonapophyses IX and gonaspiculum bridge, dorsal view **31** gonocoxa VIII and gonapophysis VIII, left view **32** sternum VII, ventral view. Scale bars: 0.2 mm.

**Figures 33–35.**
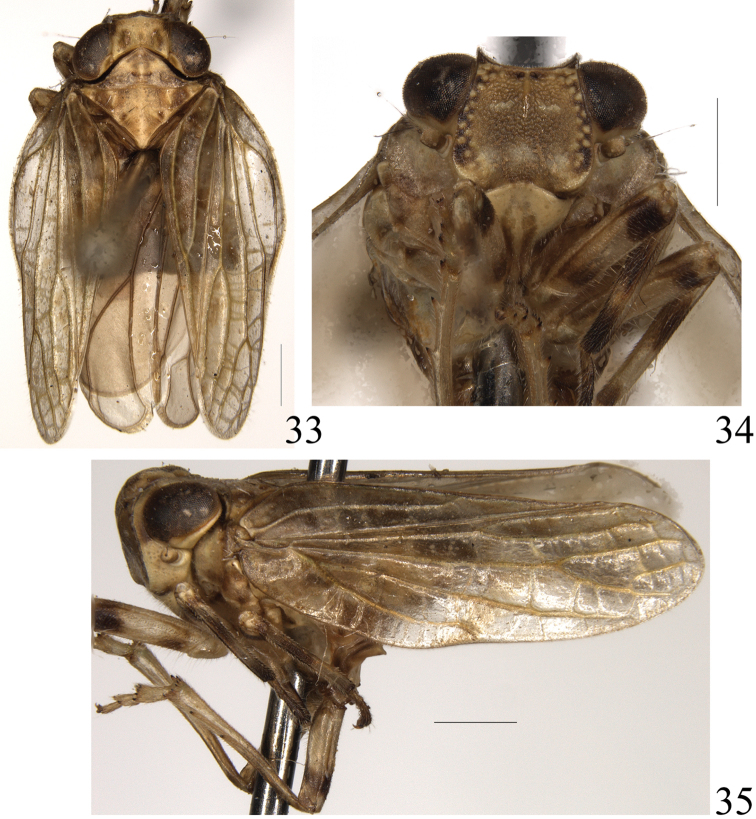
*Sarimodes parallelus* sp. n. **33** adult, dorsal view **34** frons and clypeus **35** adult, lateral view. Scale bars: 1 mm.

**Figures 36–41.**
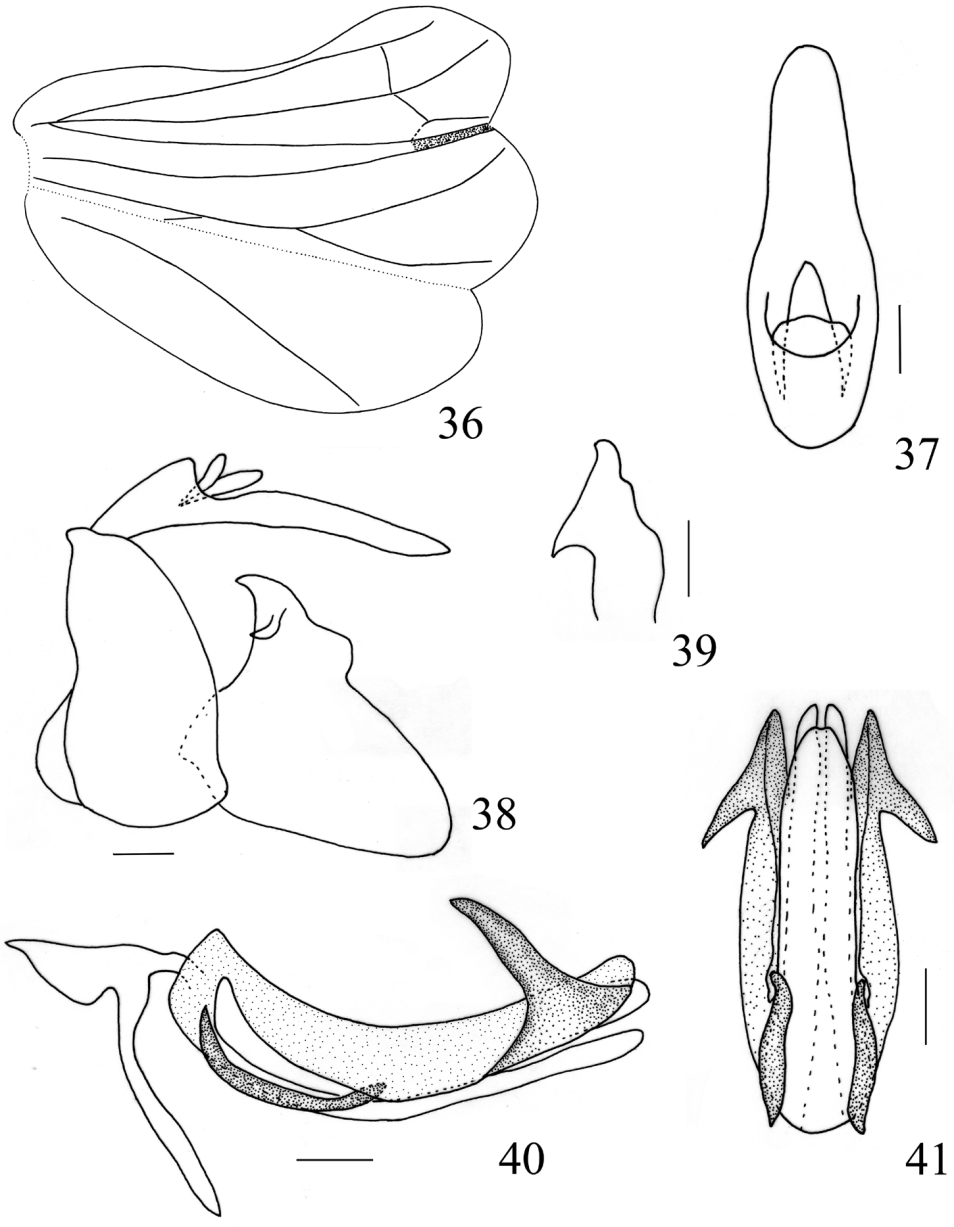
*Sarimodes parallelus* sp. n. **36** hind wing **37** male anal segment, dorsal view **38** male genitalia, lateral view **39** capitulum, dorsal view **40** phallus, left view **41** phallus, ventral view. Scale bars: 0.2 mm.

Male terminalia. Anal segment relatively long, anus stubbed, located near base of anal segment (Figs 25, 37). Pygofer in lateral view with hind margin oblique, produced near ventral margin (Figs 22, 38). Phallobase with dorsolateral lobe bearing a pair of strong and long processes near apex, directing cephalad, ventral lobe separate from dorsolateral lobe at base, narrowing to apex; aedeagus with a pair of long hooks at middle (Figs 23, 24, 40, 41).

##### Distribution.

China (Taiwan, Hainan)

#### Sarimodes clavatus sp. n.

Taxon classificationAnimaliaHemipteraIssidae

http://zoobank.org/974ADF4E-9EE5-4D0E-84A4-1F27041D9DEF

Figs 17–19 , 20–25 , 26–32

##### Type material.

Holotype: male, China, Hainan Province, Jianfengling Mountain, 14 December 1974, coll. Fasheng Li. Paratypes: 1 male, China, Hainan Province, Jianfengling Mountain, 15 June 1982, coll. Youdong Lin; 1 male, Hainan Province, Jianfengling Mountain, 24 November 1981, coll. Zhenyao Chen; 1 male, Hainan Province, Jianfengling Mountain, 900m, 10 April 1980, coll. Jiang Xiong; 1 female, Hainan Province, Jianfengling Mountain, 18 March 1982, coll. Yuanfu Liu; 1 female, Hainan Province, Jianfengling Mountain, 31 March 1984, coll. Zhiqing Chen; 1 female, Hainan Province, Limu Mountain, 27 May 1984, coll. Maobin Gu.

##### Diagnosis.

This new species resembles *Sarimodes taimokko* Mastumura, but differs from the latter by 1) frons with median carina distinct at upper half, in *Sarimodes taimokko*, frons with median carina distinct at basal third; 2) genital style with hind margin almost straight, in *Sarimodes taimokko*, genital style with hind margin weakly concave near middle; 3) phallobase with dorsolateral lobe bearing a pair of long clavate processes near apex, aedeagus with a pair of curved hooks at middle, in *Sarimodes taimokko*, phallobase with dorsolateral lobe bearing a pair of short triangular processes, aedeagus each with two processes, inner one slightly short.

##### Description.

Male length (n = 4) (including tegmen): 7.6–7.9 mm, length of tegmen: 6.6–6.9 mm; female length (n = 3) (including tegmen): 8.8–9.5 mm, length of tegmen: 7.8–9.5 mm.

***Coloration.*** Body fulvous with fuscous maculae. Vertex yellowish brown. Eyes black brown. Frons fuscous with pale tubercules at black lateral area. Clypeus brown with two dark lateral fascia. Rostrum dark brown and black at apex. Ocelli yellowish brown. Pronotum and mesonotum yellowish brown. Tegmen fulvous with fuscous and yellow speckles. Hind wing brown, veins fuscescent. Leg fulvous with fuscous transverse stripes, tips of teeth black (Figs 17–19). Abdomen fulvous and fuscous medially.

***Head and thorax.*** Vertex hexagonal, 1.2 times longer than wide in middle line, anterior margin angulately convex at middle, posterior margin deeply angulately excavate (Fig. 17). Frons slightly longer than wide, upper margin distinctly concave, with median and sublateral carinae present at upper half of frons (Fig. 18). Clypeus smooth with disc slightly convex. Rostrum reaching post-trochanter. Pronotum almost as long as vertex, with anterior margin acutely convex, posterior margin nearly straight, only shallowly emarginate at middle, median carina distinct (Fig. 17), paranotal lobe smooth, ventral margin oblique and straight, lateroventral angle subacute (Fig. 18). Tegmen elongate, 3.1 times longer than wide at widest part at basal third (Fig. 19). Hind wing with single transverse vein in between R and M and between M and CuA1 respectively; CuA2 and CuP fused from one third of CuA2 to apex, the fused part relatively thin and long (Fig. 20). Metatibiotarsal formula 2+7/9/2.

***Male terminalia.*** Anal segment cyathiform in dorsal view, 2.1 times longer than widest part, lateral margin weakly widened at base, apical margin weakly concave at middle (Fig. 25). Phallobase with dorsolateral lobe bearing a pair of long clavate processes near apex, ventral lobe with apical margin acutely convex; aedeagus with a pair of curved hooks at middle (Figs 23, 24). Genital style in lateral view subtrianglar, hind margin almost straight, caudo-ventral angle slightly convex (Fig. 22). Capitulum short and wide, with a very small lateral tooth (Fig. 21).

***Female terminalia.*** Anal segment elongate, nearly oblong in dorsal view, 2.5 times longer than widest part, apical margin slightly convex; anus short, situated at base of anal segment (Fig. 26). Gonoplac with apical margin oblique and convex at dorsal half, disc elevated near base in dorsal view, fork faintly pigmented (Figs 27, 28). Proximal part of posterior connective lamina of gonapophyses IX strongly convex in lateral view, median field single lobed, lateral fields obtusely bent at distal part (Figs 29, 30). Anterior connective laminae of gonapophysis VIII broad, ventral margin straight, bearing one tiny tooth near apex, apical group with three short stout teeth, with three keeled teeth in lateral group (Fig. 31). Sternum VII with posterior margin nearly straight at middle (Fig. 32).

##### Etymology.

The specific epithet is derived from the Latin word “clavatus”, referring to dorso-lateral lobe of phallobase having a clavate process in lateral view.

##### Distribution.

China (Hainan).

#### Sarimodes parallelus sp. n.

Taxon classificationAnimaliaHemipteraIssidae

http://zoobank.org/40B74FF0-51AE-43F8-8583-9911A2C885D3

Figs 33–35 , 36–41

##### Type material.

Holotype: male, China, Hainan Province, Jianfengling Mountain, 27 May 1983, coll. Maobin Gu.

##### Diagnosis.

This new species resembles *Sarimodes clavatus* sp. n. in the present paper, but differs from the latter by 1) frons approximately 1.25 times wider than long in middle line, in *Sarimodes clavatus*, frons slightly longer than wide; 2) genital style with hind margin produced near apex, caudo-ventral angle strongly convex, in *Sarimodes clavatus*, genital style with hind margin almost straight, caudo-ventral angle slightly convex; 3) aedeagus with a pair of hooks semicircularly curved, in *Sarimodes clavatus*, aedeagus with hooks almost straight, slightly curved dorsally at apex.

##### Description.

Male length (n = 1) (including tegmen): 6.8 mm, length of tegmen: 5.8 mm.

***Coloration.*** Generally brown with pale brown carinae and dark brown maculae. Vertex yellowish brown with black brown spots. Eyes dark brown. Frons dark brown with pale brown tubercules, near lateral and apical margins black. Gena yellowish brown with dark macula in front of eyes. Antenna with scape pale brown, pedicel brown with pale sensory pits. Clypeus yellowish brown with dark brown longitudinal stripes. Tegmen brown. Hind wing yellowish brown. Leg brown, base and apex of fore and mid femora and tibiae with dark brown band, and base of hind femora dark brown, tips of teeth black. Abdomen ventrally and dorsally brown, disc dark brown (Figs 33–35).

***Head and thorax.*** Vertex nearly hexagonal, approximately 2 times wider than long in middle line, anterior margin weakly angulately convex at middle, posterior margin distinctly obtusely concave (Fig. 33). Frons approximately 1.25 times wider than long in middle line, upper margin moderately concave, with median carina present at basal half, with a row of submarginal tubercules (Fig. 34). Pronotum narrower than head combined with eyes, longer in middle line than vertex, median carina distinct, with several small tubercules at lateral area (Fig. 33); paranotal lobes lamelliferous, with three small tubercles along posterior margin, ventral margin moderately oblique (Fig. 34). Tegmen approximately 3.2 times longer than widest part (Fig. 35). Hind wing with single transverse vein in between R and M and between M and CuA1 respectively; CuA2 thoroughly fused with CuP, the fused part relatively thick and short (Fig. 36). Metatibiotarsal formula 2+7/8/2.

***Male terminalia.*** Anal segment elliptical, 2.9 times longer than widest part near base, apical margin obtusely convex (Fig. 37). Phallobase with dorsolateral lobe bearing a pair of spiniform processes near apex, directing cephalad, ventral lobe with apical margin weakly concave medially; aedeagus with a pair of almost straight hooks at middle (Figs 40, 41). Genital style with hind margin obtusely convex near apex, caudo-ventral angle strongly convex (Fig. 38). Capitulum short, with posterior margin sinuate, apex pointed, with triangular lateral tooth (Fig. 39).

##### Etymology.

The specific epithet is derived from the Latin word “parallelus”, referring to the pair of ventral hooks of aedeagus being nearly parallel in ventral view.

##### Distribution.

China (Hainan).

## Discussion

The genus *Sarima* currently comprises 23 species including *Sarima bifurcus* sp. n., widely distributed in Oriental region, and also extending into the Eastern Palaearctic and Australian regions. Gnezdilov (2013a) proposed that the genus *Sarima* *sensu stricto* apparently endemic to Sri Lanka and that the generic position of other species described in this genus from other regions needed to be revised. However, the discovery of the new species *Sarima bifurcus* sp. n. from China (Yunnan) in the present paper shows the genus *Sarima* is not an endemic taxon of Sri Lanka. The genus *Sarima* appears to be a large group mainly widely distributed in the Oriental Region. Of course, some species of *Sarima* (*Sarima amagisana*, *Sarima ryukyuana*, *Sarima tappana*) need further study (Gnezdilov 2013b) and the genus *Sarima* needs to be revised.

The genus *Sarima* is very close to the genus *Eusarima* according to the similar structure of phallus, phallobase with dorsolateral lobe split near apex, lateral lobe forming a small short process directing caudad, and aedeagus with long process arising from subapex. But *Eusarima* in contrast to the *Sarima* has the frons with clear sublateral carinae, tegmen without hypocostal plate and vein MP branched at middle. The genus *Sarima* is also close to the genus *Sarimodes* by the similar veins on tegmen. But the genus *Sarimodes* has the frons with short median carina, tegmen without hypocostal plate, and phallobase with dorsolateral lobe bearing a process directing cephalad laterally. The phylogenetic relationships of these close genera needs further study.

## Supplementary Material

XML Treatment for Sarima

XML Treatment for Sarima bifurcus

XML Treatment for Sarimodes

XML Treatment for Sarimodes clavatus

XML Treatment for Sarimodes parallelus
